# Ovulatory Response of Weaned Sows to an Altered Ratio of Exogenous Gonadotrophins

**DOI:** 10.3390/ani10030380

**Published:** 2020-02-26

**Authors:** Rodrigo Manjarín, Jose Carlos García, Lia Hoving, Nicoline M. Soede, Magdalena Maj, Juan Carlos Dominguez de Tejerina, Roy N. Kirkwood

**Affiliations:** 1 Animal Science Department, California Polytechnic State University, San Luis Obispo, CA 93407, USA; rmanjari@calpoly.edu; 2 Department of Medicine, Surgery and Veterinary Anatomy, University of León, 24004 Leon, Spain; ctijgg@unileon.es (J.C.G.); jcdomt@unileon.es (J.C.D.d.T.); 3 Department of Animal Sciences, Wageningen University, Wageningen PB 9101, 6700, The Netherlands; Lia.Hoving@varkenski.nl (L.H.); nicoline.soede@wur.nl (N.M.S.); 4 Biological Sciences Department, California Polytechnic State University, San Luis Obispo, CA 93407, USA; mmaj@calpoly.edu; 5 School of Animal and Veterinary Sciences, University of Adelaide, Roseworthy, SA 5005, Australia

**Keywords:** sows, gonadotrophins, follicle, ovulation

## Abstract

**Simple Summary:**

Efficient pork production relies on a consistent supply of market pigs. To achieve breeding targets, gonadotrophins can be administered at weaning to stimulate estrus onset. The present study examined the impact of supplemental human chorionic gonadotrophin activity (i.e., hCG), during a follicular phase induced by a standard gonotrophin protocol (i.e., PG600), in both ovarian follicular development and fertility in multiparous sows. The results confirmed that supplemental hCG at 24 h after PG600 increased follicle growth and reduced the interval to ovulation, but also increased the incidence of follicle cysts and reduced pregnancy success.

**Abstract:**

At weaning, 33 mixed parity Hypor sows received either an injection of 400 IU equine chorionic gonadotrophin and 200 IU human chorionic gonadotrophin (hCG) (PG600; *n* = 13), PG600 with an additional 200 IU hCG 24 h later (Gn800; *n* = 11), or served as non-injected controls (*n* = 9). All gonadotrophin treated sows received an injection of 750 IU hCG at 80 h after weaning to induce ovulation (designated as time 0 h). At 0, 24, 36, 40, 44, 48, and 60 h, all sows were subject to transrectal ultrasonography to determine numbers and sizes of large (>6 mm) follicles and time of ovulation. The interval from injection of 750 IU hCG to ovulation was shorter in Gn800 compared to PG600 sows (*p* = 0.02), and more Gn800 sows had ≥9 preovulatory follicles compared to PG600 and controls (*p* = 0.02 and 0.003, respectively). Follicular cysts were evident in both PG600 and Gn800 sows.

## 1. Introduction

In commercial practice, pig production is constrained by the availability of sows with a predictable and synchronous post-weaning estrus [1]. In addition to accumulating non-productive days, if breeding targets are missed empty farrowing crates result. To minimize missed breeding targets, gonadotrophins can be administered at weaning to stimulate estrus onset. The gonadotrophin preparation most often used to stimulate estrus in female pigs is PG600^®^ (Merck Animal Health, Readington Township, NJ, USA), which is a combination of 400 IU equine chorionic gonadotrophin (eCG) and 200 IU human chorionic gonadotrophin (hCG). The eCG primarily has follicle-stimulating hormone (FSH)-like activity, although a variable luteinizing hormone (LH)-like activity is also observed, while hCG is an LH analogue. While both FSH and LH activity are responsible for early follicle development, the principal driver of ovarian follicle development beyond 4 mm to ovulation in female pigs is LH [2]. Confirming this, we previously observed that when gilts pre-treated with FSH received an injection of hCG, the estrus and ovulation responses were improved compared to FSH-treated gilts that received eCG [3].

More recently, it has been observed that, in comparison to both untreated and PG600-treated primiparous sows, an additional 200 IU hCG injected concurrent with PG600 increased numbers of sows exhibiting estrus by 7 d after weaning; both gonadotrophin treatments increased farrowing rates [4]. Numbers of ovulations can be dose-dependently increased by eCG [5], although eCG-induced superovulation is also associated with an increase in poorer quality embryos [6]. Further, it was demonstrated that injection of an additional 200 IU hCG at 24 h after PG600 resulted in a reduced farrowing rate in multiparous sows but, interestingly, not in younger sows [7]. The present study was undertaken to monitor sow ovarian follicular dynamics, and so gain insight into why the fertility of multiparous sows may decrease when an additional 200 IU hCG is injected at 24 h after PG600.

## 2. Material and Methods

### 2.1. Animals and Experimental Design

This study was performed on a 1000-sow farrow-to-wean facility near Leon, Spain. The University of León Animal Care Committee reviewed and approved the protocol and procedures. Animals were cared for in accordance with local animal care guidelines. Mixed parity (4.6 ± 1.2) Hypor sows (*n* = 33) were weaned into individual gestation stalls and were fed 2.5 kg/d of a wheat and soybean meal diet formulated to provide 13% crude protein and 13.1 MJ metabolizable energy/kg. At weaning, sows were allotted by parity to receive an injection of PG600® (Gn600; *n* = 13), PG600^®^ followed by 200 IU hCG (Chorulon^®^, Merck) at 24 h (Gn800; *n* = 11) or served as non-injected controls (Control; *n* = 9). Fence-line boar exposure was provided for 5 min once daily from 3 d after weaning to facilitate estrus detection. Those sows treated with PG600 at weaning received an injection of 750 IU hCG at 80 h after weaning (designated as time 0 h) in order to induce ovulation. These sows were subjected to transrectal real-time ultrasonography of the left ovary at 0, 24, 36, 40, 44, 48, and 60 h using a 7.5 MHz multi-angle mechanical probe (Scanner 200, Pie Medical, Maastricht, The Netherlands) to determine follicular number and size. Time of ovulation was deemed to be the time midway between an observed absence of preovulatory follicles (>6 mm) and the previous examination [8]. Follicles >12 mm at the final scan were deemed to be cystic. 

### 2.2. Chemical Analysis

To confirm ovulation, blood samples were obtained from all sows via jugular venipuncture at 9 and 19 d after weaning and analysed for progesterone concentrations in a single assay using a commercial enzyme-linked immunosorbent assay kit (Immulite, Siemens Medical Solutions Diagnostics, Tarrytown, NY, USA). Serum was harvested within 4 h by centrifugation at 2000× *g* at 4 °C for 15 min. Assay sensitivity and intra-assay coefficient of variation were 0.2 ng/mL and 8.1%, respectively. An increase in progesterone concentrations from <1 ng/mL on day 9 to ≥5 ng/mL on day 19 was considered to indicate ovulation. Sows were artificially inseminated with commercially-derived semen doses containing 3 × 10^9^ motile sperm at detection of estrus and again at 24 h. Sows were allowed to go to term to determine farrowing rates.

### 2.3. Statistical Analyses

The incidence of estrus and ovulation, number of ovaries with ≥9 ovulatory follicles and follicular cysts, and farrowing rates were analyzed by logistic regression using a generalized linear mixed model in SAS (PROC GLIMMIX; SAS 9.2, SAS Institute Inc., Cary, NC, USA) that included treatment as fixed effect and parity as covariate. Normality of the residuals and presence of outliers were assessed by PROC UNIVARIATE using the Shapiro-Wilk test, Q-Q-plots and externally studentized residuals. Data were power transformed by a parameter φ whose optimal value was estimated using the maximum likelihood (ML) method [9]. Pairwise comparisons were analyzed by Student’s *t*-tests. Data are presented as probabilities and least square means ± standard error. Significant effects were considered at *p* ≤ 0.05.

## 3. Results

All sows showed estrus by day 4 post-weaning, and only one sow in the PG600 group failed to ovulate (Table 1). Interval from injection of 750 IU hCG to ovulation was shorter in Gn800 compared to PG600 sows (*p* = 0.02). Number of sows with ≥9 preovulatory follicles was higher in the Gn800 group compared to PG600 and control groups (*p* = 0.02 and 0.003, respectively). Follicular cysts were evident in both PG600 and Gn800 sows with no difference in numbers between groups. Fewer Gn800 sows farrowed than PG600 sows, and 3 of 4 Gn800 sows that failed to farrow had multiple follicle cysts.

## 4. Discussion

This study assessed the effects of supplemental LH-like activity during a follicular phase induced by PG600. Results indicate that supplemental hCG at 24 h after PG600 increased follicle growth and reduced the interval to ovulation subsequent to injection of an ovulatory dose of hCG, although the incidence of follicle cysts increased with attendant reduced pregnancy success. The mechanism whereby the ovary was apparently made more responsive to an ovulatory signal is not known. The hCG can be expected to increase production of follicular testosterone which, in turn, may increase circulating FSH concentrations [10]. Higher FSH levels in association with the increased LH activity from the hCG may be associated with the more rapid follicular growth and, presumably, follicular maturation.

The gonadotrophin treatment caused some sows to develop multiple ovarian cysts. Similarly, Breen et al. [11] observed higher rates of ovarian cysts when the dose of PG600 was increased by 50% in weaned sows. It is possible that an excess of hCG down-regulated LH receptors in some follicles which could have the effect of inhibiting their response to the ovulatory LH surge, resulting in development of follicular cysts. In this regard, and although speculative, the different responses of younger and older sows may reflect a difference in endogenous circulating LH. Under conditions of lower LH, such as may occur in young sows or in association with seasonal infertility, the additional hCG would be expected to prolong the LH-like activity and improve follicular responses [4]. In contrast, if endogenous LH levels are adequate, receptor down-regulation may be more likely. In the present study, the estrus response of control sows indicates no evident background infertility, suggestive of normal circulating LH levels.

It is evident that hCG supplementation subsequent to PG600 increased the number of ovulatory follicles in weaned sows. However, this protocol is not indicated for control of estrus and ovulation on commercial units due to follicle cyst formation and an associated reduction in fertility. The impact of additional hCG concurrent with PG600 in multiparous sows remains to be determined.

## Figures and Tables

**Table 1:** Effect of human chorionic gonadotrophin (hCG) supplementation on estrus and ovulatory response, ovarian characteristics, farrowing rates, and subsequent litter size in PG600-treated weaned sows.

Item	PG600	PG800 ^c^	Control
No. Sows	13	11	9
Parity	4 ± 2.0	3.9 ± 2.7	3 ± 1.6
Sows estrous	13	11	9
Sows ovulating	12	11	9
Interval to ovulation ^d^	47.1 ± 2.1 ^a^	38.7 ± 2.3 ^b^	NA
Sows with ≥9 ovulatory follicles ^e^	3 ^a^	10 ^b^	1 ^a^
Sows with follicular cysts ^f^	3	4	0
Sows with >1 cysts/ovary	1	3	0
Progesterone >5 ng/mL d19	12	11	9
Sows farrowing (%)	12 (92.3)	7 (63.6)	7 (77.8)

^a,b^—Means with different superscripts differ at *p* ≤ 0.05; ^c^—200 IU hCG (Chorulon) injected 24 h after PG600 injection; ^d^—Interval from injection of 750 IU hCG to ovulation; ^e^—Follicles >6 mm.; ^f^—Follicles >12 mm.
